# Intranasal immunization with a helper-dependent adenoviral vector expressing the codon-optimized fusion glycoprotein of human respiratory syncytial virus elicits protective immunity in BALB/c mice

**DOI:** 10.1186/1743-422X-10-183

**Published:** 2013-06-07

**Authors:** Yuan-Hui Fu, Jin-Sheng He, Wei Qiao, Yue-Ying Jiao, Ying Hua, Ying Zhang, Xiang-Lei Peng, Tao Hong

**Affiliations:** 1 College of Life Sciences & Bioengineering, Beijing Jiaotong University, 3 Shangyuan Residence, Haidian District, Beijing 100044, China; 2 Department of Immunology, Anhui Medical University, Hefei, Anhui 230032, China; 3 Institute for Viral Disease Control and Prevention, Chinese Center for Disease Control and Prevention, Beijing 100052, China

**Keywords:** Human respiratory syncytial virus, Helper-dependent adenovirus vectors, Fusion glycoprotein, Protective immunity, Immune responses

## Abstract

**Background:**

Human respiratory syncytial virus (RSV) is a serious pediatric pathogen of the lower respiratory tract. Currently, there is no clinically approved vaccine against RSV infection. Recent studies have shown that helper-dependent adenoviral (HDAd) vectors may represent effective and safe vaccine vectors. However, viral challenge has not been investigated following mucosal vaccination with HDAd vector vaccines.

**Methods:**

To explore the role played by HDAd as an intranasally administered RSV vaccine vector, we constructed a HDAd vector encoding the codon optimized fusion glycoprotein (Fsyn) of RSV, designated HDAd-Fsyn, and delivered intranasally HDAd-Fsyn to mice.

**Results:**

RSV-specific humoral and cellular immune responses were generated in BALB/c mice, and serum IgG with neutralizing activity was significantly elevated after a homologous boost with intranasal (i.n.) application of HDAd-Fsyn. Humoral immune responses could be measured even 14 weeks after a single immunization. Immunization with i.n. HDAd-Fsyn led to effective protection against RSV infection on challenge.

**Conclusion:**

The results indicate that HDAd-Fsyn can induce powerful systemic immunity against subsequent i.n. RSV challenge in a mouse model and is a promising candidate vaccine against RSV infection.

## Introduction

Human respiratory syncytial virus (RSV) is a serious pediatric pathogen of the lower respiratory tract [1,2] and causes significant illness in the elderly and adults with underlying risk factors such as immunodeficiency [3,4]. Several approaches have been used to develop vaccines against RSV infection including live/attenuated vaccines, viral and bacterial vector vaccines, DNA vaccines, and adjuvant subunit vaccines. Although three vaccine candidates, two live attenuated and a viral vector vaccine, have been evaluated in seronegative infants and seropositive children, respectively [1,5], no RSV vaccines have been approved for use in humans [3,4,6].

The replication-deficient first-generation adenovirus serotype 5 (FGAd5) vector can be readily grown and purified in large quantities and is able to express high levels of the transgene in dividing and non-dividing cells. Therefore, it is considered to be an attractive vaccine vector. Studies have shown that FGAd-based vaccines can elicit robust protective immune responses against RSV infection and represent promising candidates for an RSV vaccine [7-9]. However, the majority of the human population, particularly in developing countries and in adult populations, has been exposed to Ad5 and has pre-existing neutralizing antibodies that can attenuate Ad5-vector vaccine delivery and efficacy [10,11]. For Ad5-vector vaccines against RSV infection, the situation is somewhat different. Ad5-vector vaccines encoding RSV antigens can be administered in Ad5-seronegative children aged between 6 and 24 months, when maternal antibodies have declined substantially and a rapid increase in Ad5 seroprevalence from natural infections is absent [11]. It would also have a major impact on morbidity by preventing a first or second RSV infection in these infants [1].

In contrast to the FGAd vector, the helper-dependent adenoviral (HDAd) vector has all the adenovirus (Ad) coding regions deleted and exhibits lower Ad-specific cellular immunity and stronger longer-term gene expression *in vivo*[12-14]. Therefore, HDAd vectors have been constructed as a safer vector platform with increased transgene capacity compared to FGAd vectors [12-14]. For efficient packaging, the HDAd must often include “stuffer” DNA. The choice of stuffer DNA is important with regard to vector stability and replication efficiency. In general, noncoding eukaryotic DNA is preferable while repetitive elements and unnecessary homology with the helper virus should be avoided. Recent data from our group and others have demonstrated that HDAd vectors are capable of generating stronger immune responses against transgenes such as reporter proteins and HIV-env than FGAd vectors in mice [12-15]. However, there has been no investigation of viral challenge following mucosal vaccination with HDAd vector vaccines.

In this study, mice were immunized intranasally with a HDAd vector containing the codon-optimized full-length fusion glycoprotein (Fsyn) of RSV. The animals were then monitored for induction of RSV-specific humoral and cellular immune responses and protection against i.n. RSV challenge. We report that the HDAd-Fsyn vaccine candidate induces both a serum antibody response and an RSV-specific CD8^+^ T-cell response. To the best of our knowledge, this is the first study showing that an i.n. HDAd vector vaccine is highly effective at stimulating RSV F-specific humoral and cellular immunities. Our findings will be useful in the development of vaccines against RSV infection.

## Materials and methods

### Preparation and titration of RSV stock

Subgroup A RSV Long strain (kindly provided by Prof. Y. Qian, Capital Institute of Pediatrics, Beijing, China) was propagated in HEp-2 cells (ATCC, Rockefeller, MD, USA) in Dulbecco’s modified Eagle medium (DMEM; Invitrogen, Carlsbad, CA, USA) supplemented with 2% fetal calf serum (Invitrogen), L-glutamine (2 mol/L), penicillin G (40 U/mL), streptomycin (100 μg/mL) and 0.2% sodium bicarbonate. The infectivity of the resulting RSV was titrated using the immunoenzyme assay described by Wang et al. [16] with slight modifications.

### Construction and purification of HDAd-Fsyn

For construction of HDAd-Fsyn and improving the expression level of F gene, the F sequence was codon optimized and synthesized by Geneart (Regensburg, Germany) (GenBank database entry EF566942 [8]). The Fsyn gene expression cassette with the cytomegalovirus (CMV) enhancer/promoter and the bovine growth hormone (BGH) polyA was cloned into the HDAd shuttle plasmid pSC11[17]. The expression cassette was digested with restriction enzyme I-*Sce*I and I-*Ceu*I and cloned into the HDAd backbone plasmid pSC15B [17] to produce pSC15B-Fsyn. The resulting plasmid was linearized using the restriction enzyme *Pme*I and transfected into 293Cre4 cells (293 cells expressing Cre recombinase; Microbix, Toronto, Canada) using the calcium phosphate transfection method. The cells were infected with E1-E3-deleted helper virus H14 (Microbix) 16 h after transfection. HDAd-Fsyn was amplified by serial coinfection of 293Cre4 cells with helper virus and crude lysates from the previous passage. Large-scale production of HDAd-Fsyn was achieved by infecting 293Cre4 in 150-mm dishes with crude HDAd-Fsyn and helper virus, purified by CsCl banding. The concentration of purified HDAd-Fsyn was determined using a previously described method [18]. The amount of contaminated helper virus was also ascertained by determining the number of plaque-forming units (pfu/mL) after titration of the purified HDAd-Fsyn in 293 cells [18,19]. HDAd vector encoding enhanced green fluorescent protein (HDAd-EGFP) was constructed as previously described and used as a control vector [15].

### Animals

Specific pathogen-free female BALB/c mice, aged between 6 and 8 weeks, were purchased from Vital River Laboratories (Beijing, China) and kept under specific pathogen-free conditions. All animal studies were performed according to the guidelines of our Institutional Animal Care and Use Committee. The protocol was approved by the Committee on the Ethics of Animal Experiments of Beijing Jiaotong University (Permit number: 2010–0013). All surgeries were performed under sodium pentobarbital anesthesia, and all efforts were made to minimize suffering.

### Immunization and challenge

BALB/c mice were divided into four groups: HDAd-Fsyn (once), HDAd-EGFP (once), HDAd-Fsyn (prime + boost), and HDAd-EGFP (prime + boost). After BALB/c mice being lightly anesthetized with pentobarbital sodium (4 μg/kg weight), a dose of 5×10^8^ virus particles (in 50 μL) of either HDAd-Fyn or HDAd-EGFP was delivered intranasally either once (in week 0) or twice (in weeks 0 and 4). Three weeks after the final immunization, mice that received two doses of HDAd-Fsyn or HDAd-EGFP were subjected to i.n. challenge with 100 μL of subgroup A RSV Long strain (10^6^ pfu/mL).

### Collection of splenocytes

Spleens from vaccinated mice were harvested and placed in mouse lymphocyte separation medium. After BALB/c mice being lightly anesthetized with pentobarbital sodium (4 μg/kg weight), the mice were were sacrificed by cervical dislocation. The spleens were triturated and passed gently through cell strainers (Becton-Dickinson, San Jose, CA, USA) to obtain single-cell suspensions, which were centrifuged at 800*g* for 30 min. The splenocytes were collected and washed with complete RPMI 1640 medium (Invitrogen).

### Preparation of lung homogenates

After sacrifice, the left lung was harvested from vaccinated mice in each group. Each lung was washed three times in phosphate-buffered saline (PBS), weighed, placed in PBS (1 ml per 0.1 g of tissue) containing 0.1% bovine serum albumin (BSA; Huashengyili, Beijing, China), and homogenized with a glass tissue grinder. The homogenates were centrifuged (10,000*g* for 5 min) and the supernatant was analyzed for IgA by ELISA as previously described [15].

### Analysis of RSV-F-specific antibody production

Blood was obtained from the retro-orbital plexus using a capillary tube and collected in an Eppendorf tube. After centrifugation (5000*g* for 15 min), sera were stored at −20°C. RSV-F-specific antibody responses in immunized mice were measured by ELISA as previously described [7,20]. In brief, RSV was adsorbed onto ELISA plates overnight in carbonate buffer (pH 9.8) at 4°C. The plates were blocked with 5% fetal bovine serum (FBS) in PBS for 2 h at 37°C. After thorough washing with 1% BSA in PBS, serum or homogenate was added to the plate and incubated for 1 h at 37°C. After further washing, the plates were incubated for 1 h with horseradish peroxidase (HRP)-conjugated anti-mouse IgA (1:500 dilution), IgG1 (1:5000 dilution), IgG2a (1:5000 dilution), or IgG (1:5000 dilution) antibodies (Santa Cruz). The plates were developed with 100 μl of tetramethyl benzidine (TMB; Sigma, St. Louis, MO, USA) substrate solution, stopped with 50 μl of 2 mol/L H_2_SO_4_, and analyzed at 450 nm using an ELISA plate reader (Tecan, Grödig, Austria).

### RSV-specific neutralizing antibody assay

To analyze the RSV-specific neutralizing antibody titer, serum samples were heat-inactivated at 56°C for 30 min. Serial two-fold dilutions of the mouse sera were prepared in virus diluent (Eagle’s minimal essential medium with L-glutamine containing 2% FBS, 2.5% HEPES (1 mol/L) and 1% antibiotic/antimycotic). To each serial diluted sample, 50 pfu of RSV virus suspension was added and incubated at 37°C for 1 h. RSV-specific neutralizing antibody titers were analyzed using the immunoenzyme assay described above. Neutralization titers are expressed as the reciprocal of the serum dilution giving a 50% reduction in pfu number in control wells.

### CD8^+^ T cell responses to RSV F

To determine the number of cytokine-producing cells, IFN-γ was measured using an ELISPOT kit (BD Biosciences, San Diego, CA, USA) according to the manufacturer’s instructions. In brief, 2×10^5^ freshly isolated splenocytes were added to each of three replicate wells coated with purified anti-mouse IFN-γ monoclonal antibody and stimulated with peptides F85–93 and F249–258 (10 μg/mL), corresponding to two known H-2 K^d^-restricted RSV F protein epitopes (KYKNAVTEL [21] and TYMLTNSEL [22], respectively; purity ≥95%), for 24 h at 37°C in a 5% CO_2_ incubator. Unstimulated splenocytes were used to measure background cytokine production. The cells were then lysed with deionized water and the plates were incubated at room temperature with biotinylated IFN-γ antibody for 2 h and peroxidase-labeled streptavidin for 1 h. After washing with PBS, 100 μL of the final substrate solution (BD Biosciences) was added to each well and spot development was monitored. The plates were washed with distilled water to terminate the reaction. IFN-γ spot-forming cells (SFCs) were counted automatically using a CTL ELISPOT reader (BD Biosciences) and analyzed using ImmunoSpot image analyzer software v4.0 (BD Biosciences).

### RSV titer in lungs

Mice were sacrificed on day 5 after the challenge. The left lung was harvested from mice in each group, weighed, placed in sterile stabilizing buffer (1 ml/g lung), and homogenized with a glass tissue grinder. The homogenates were centrifuged (10,000*g* for 1 min) and the RSV titers in the supernatants were measured by immunoenzyme assay as follows. HEp-2 cells (2×10^4^ cells/well) were added to 96-well tissue culture plates (Corning Incorporated, Corning, USA) and incubated overnight at 37°C After removal of the medium, serial ten-fold dilutions of the clarified homogenate supernatants were adsorbed onto cell monolayers that had been washed with DMEM without serum in duplicate. Plates were incubated at 37°C for 45 min, the supernatant was removed, and 100 μl of DMEM containing 1.0% carboxymethyl cellulose (Sigma) was added to each well. After 3 days of incubation at 37°C, the overlay was removed, the cells were washed with PBS, and the monolayers were fixed with 1 ml of cold 95% alcohol for 10 min. After washing, plates were blocked in 5% nonfat dry milk in PBS for 1 h at RT. Anti-F monoclonal antibody (1:200 dilution) was added to the wells and incubated for 2 h, followed by 1 h incubation with HRP-conjugated anti-mouse IgG antibodies (1:5000 dilution in 5% nonfat milk; Santa Cruz) at 37°C. After washing twice with PBS containing 0.5% Tween 20 (Sigma), the plates were developed with 100 μl of TMB substrate solution. Individual plaques were counted under an inverted microscope and expressed as pfu.

### Statistical analyses

Statistical analyses were performed using SPSS 11.5 software (SPSS, Chicago, IL, USA). Differences were compared using the Tukey test. *P*<0.05 was considered statistically significant.

## Results

### Characterization of antibody responses induced by HDAd-Fsyn

**Figure 1:**
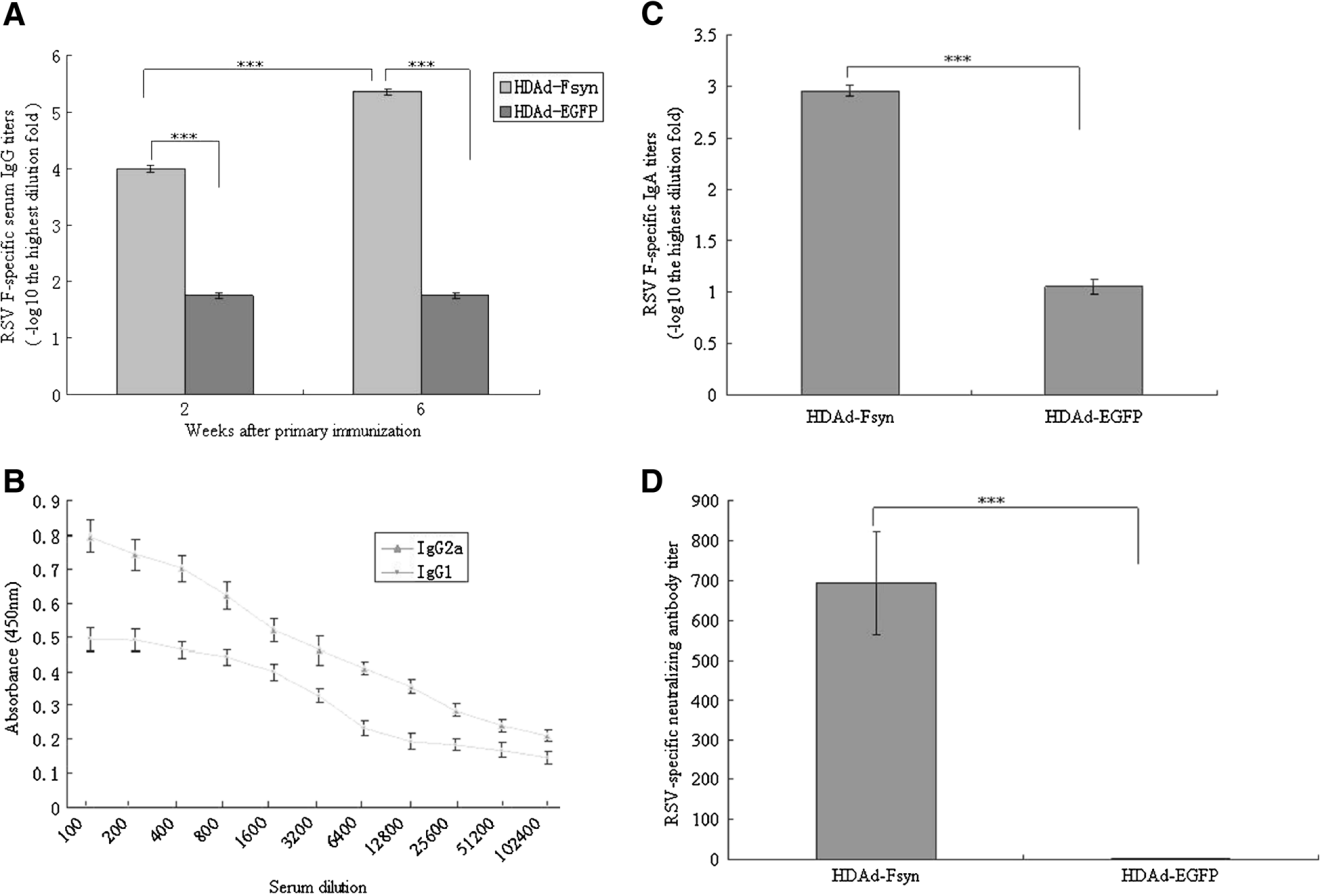
**Characterization of antibody responses following intranasal administration of HDAd-Fsyn in BALB/c mice.** Six BALB/c mice were immunized intranasally with HDAd-Fsyn in weeks 0 and 4. ***P<0.0001. Values represent mean ± SEM. (**A**) Serum anti-RSV F IgG responses induced by HDAd-Fsyn. RSV F-specific antibody titers were measured by ELISA in weeks 2 and 6 after primary immunization. The results represent log_10_ endpoint values for six individual mice. (**B**) IgG subtype in sera from HDAd-Fsyn-immunized mice. Sera were obtained in week 6 after primary immunization and ELISA was performed as described in Materials and Methods. Results represent absorbance (450 nm) for samples from six individual mice. (**C**) Anti-RSV F IgA levels induced by HDAd-Fsyn in lung homogenates. RSV F-specific IgA antibody titers were measured by ELISA in week 6 after primary immunization. Results represent log_10_ endpoint values for six individual mice. (**D**) Virus-neutralizing activity in sera from animals vaccinated with HDAd-Fsyn. Virus-neutralizing antibody titers were analyzed by immunoenzyme assay in week 6 after primary immunization. Results are expressed as neutralizing titers corresponding to the serum dilution giving 50% inhibition of plaque formation.

Serum antibody responses play vital roles in protection against RSV infection [1]. We first examined the ability of HDAd-Fsyn to elicit RSV-F-specific serum antibody responses *in vivo*. As shown in Figure 1A, i.n. immunization with HDAd-Fsyn stimulated strong serum IgG responses. The IgG1 and IgG2a responses were also determined. Sera from mice immunized with two doses of HDAd-Fsyn showed high levels of IgG2a antibodies (Figure 1B). We also measured levels of anti-RSV IgA in the lungs following HDAd-Fsyn vaccination. RSV-F-specific IgA levels in lung homogenates were analyzed by ELISA. As shown in Figure 1C, i.n. immunization with HDAd-Fsyn generated strong IgA responses. It is known that neutralizing antibodies in serum are highly related to protection against RSV infection in the lower respiratory tract. We therefore measured the neutralizing activity in sera by immunoenzyme assay. Sera from mice immunized with HDAd-Fsyn displayed RSV-specific neutralizing activity (Figure 1D).

### Induction of cellular immune responses

**Figure 2:**
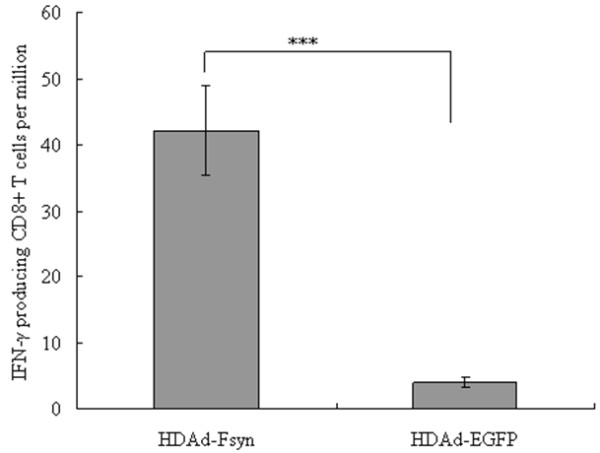
**CD8**^**+ **^**T-cell responses after immunization with HDAd-Fsyn.** Six BALB/c mice were immunized intranasally with HDAd-Fsyn in weeks 0 and 4. RSV F-specific CD8^+^ T-cell responses were assessed using ELISPOT in week 6 after the primary immunization. Results are expressed as the average number of IFN-γ-producing CD8^+^ T cells per million input splenocytes. ***P<0.0001. Values represent mean ± SEM.

It is universally recognized that RSV-specific CD8^+^ T-cell responses play a major role in virus clearance [1] and FGAd vector vaccines are well known for their ability to evoke strong cytotoxic T-lymphocyte responses [7]. Therefore, we investigated whether HDAd-Fsyn could generate IFN-γ-producing CD8^+^ T cells after *in vitro* stimulation with two known H-2 K^d^-restricted RSV F protein epitopes. RSV-F-specific CD8^+^ T-cell immune responses induced by HDAd-Fsyn were evident (Figure 2) compared to the control group, indicating that i.n. HDAd vector vaccines are competent in producing humoral and cellular immune responses against the transgene. This finding provides valuable support for our previous report [15].

### Protective efficacy of HDAd-Fsyn against RSV challenge

**Figure 3:**
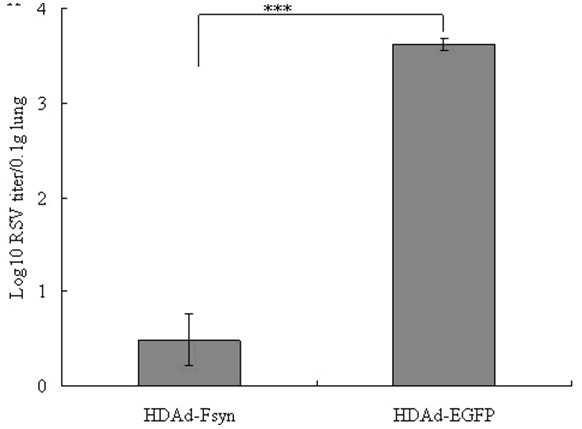
**HDAd-Fsyn induced protective immunity against intranasal RSV challenge.** Six BALB/c mice were immunized intranasally with HDAd-Fsyn in weeks 0 and 4 and then challenged 3 weeks after the final immunization with intranasal administration of 100 μl of subgroup A RSV Long strain (10^6^ pfu/ml). *P<0.05; ***P<0.0001. Values represent mean ± SEM.

To evaluate the protective efficacy of HDAd-Fsyn vaccines *in vivo*, vaccinated mice were challenged with i.n. RSV 3 weeks after the booster immunization. Five days after the challenge, when the viral load peaked in the lungs, the mice were sacrificed and lung homogenates were prepared. Compared to the control group, HDAd-Fsyn prevented RSV replication and significantly reduced viral load in the lungs. As shown in Figure 3, lung viral titers in the HDAd-Fsyn immunized group were significantly lower (3.5 ± 0.67 pfu/0.1 g lung) than in the control group (4150 ± 233.45 pfu/0.1 g lung).

### Durability of antibody responses after immunization

**Figure 4:**
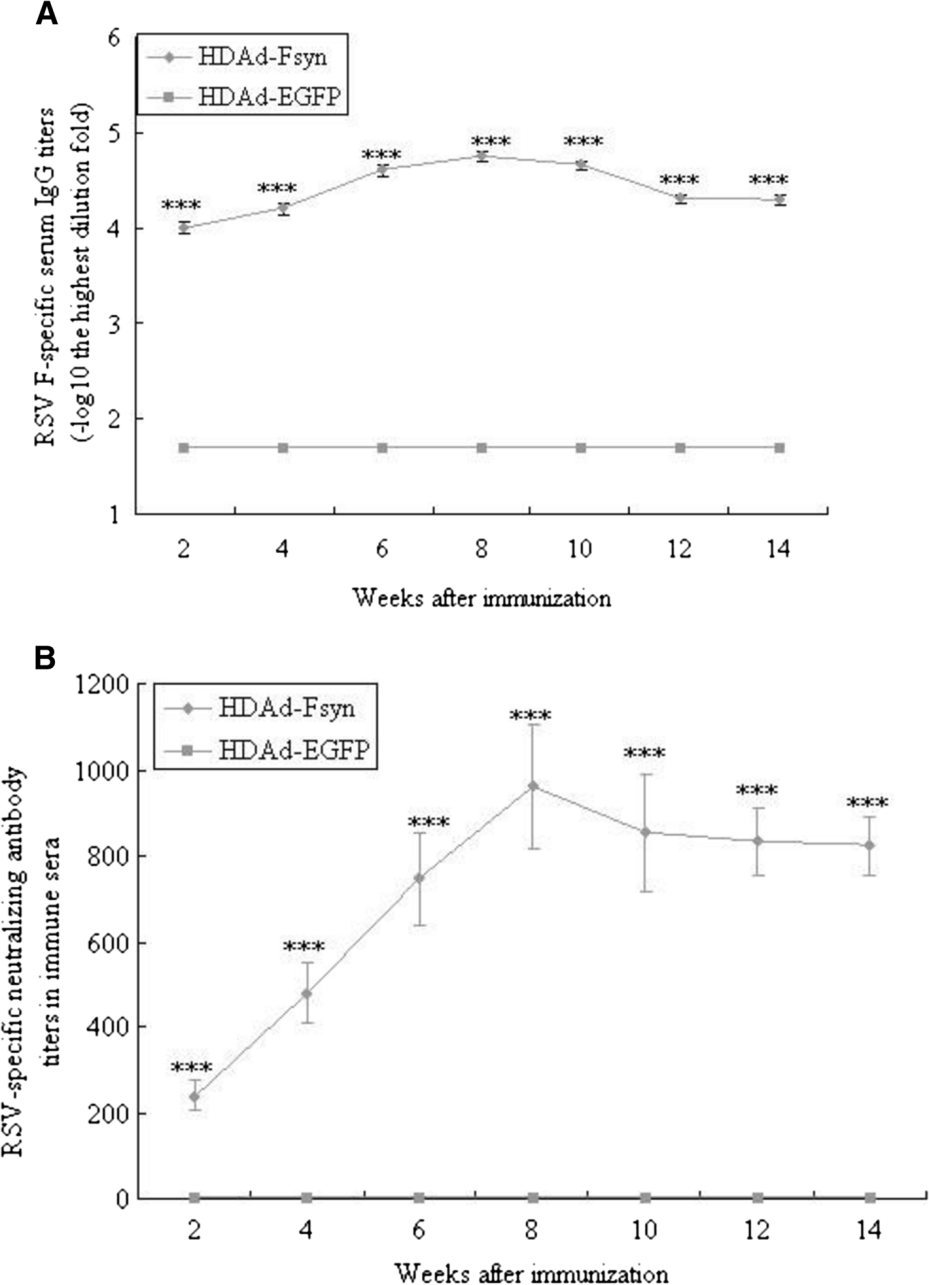
**Duration of the immune responses stimulated by single intranasal administration of HDAd-Fsyn in BALB/c mice.** Six BALB/c mice were immunized intranasally with one dose of HDAd-Fsyn in week 0. *P<0.05; ***P<0.0001. Values represent mean ± SEM. (**A**) Serum anti-RSV F IgG induced by HDAd-Fsyn. RSV F-specific antibody titers were measured by ELISA at 2-week intervals up to 14 weeks after immunization. The results represent log_10_ endpoint values for six individual mice. (**B**) Virus-neutralizing activity in sera from mice immunized with HDAd-Fsyn. Virus-neutralizing titers in sera were measured by immunoenzyme assay at 2-week intervals up to 14 weeks after immunization. Results are expressed as neutralizing titers corresponding to the serum dilution giving 50% inhibition of plaque formation.

One hallmark of a successful vaccine is induction of long-lasting protective immunity. To determine the duration of immune response induced by HDAd-Fsyn, we examined the humoral immune responses at 2-week intervals up to 14 weeks following single vaccination with HDAd-Fsyn. Both specific IgG titers (Figure 4A) and neutralization titers (Figure 4B) increased through to week 8 and then declined somewhat but remained at high levels.

## Discussion

Several studies have shown that HDAd vectors are superior to FGAd vectors for vaccine development in terms of their ability to produce immune responses against transgenes and are a robust platform for vaccination [12-14]. However, as pioneering explorations, most of the studies only investigated humoral and cellular immune responses induced by HDAd vector vaccines. There is only one report involving rectal HIV challenge following intramuscular administration of a HDAd vector vaccine encoding the envelope antigen [14]. This situation prompted us to explore the protective effect following mucosal vaccination with HDAd vector vaccine. Our study provides the first demonstration of *in vivo* immunogenicity and efficacy of i.n. HDAd-Fsyn vaccination. The HDAd-Fsyn mucosal vaccine induced long-lasting RSV-F-specific cellular and humoral immune responses and protected against RSV infection in BALB/c mice on challenge. The reduction in viral load observed was approximately 1100-fold after prime–boost immunization in mice after i.n. HDAd-Fsyn vaccination when compared with the control group. This indicates that the HDAd vector is a very efficient and convenient mucosal vaccine platform in a murine model.

A few studies have addressed stimulation of high-titer antibodies against transgenes by HDAd vector vaccines [15]. However, none of the studies investigated the neutralizing activity of these antibodies [12-14]. The presence of serum neutralizing antibodies is vital in protecting against viral infection. Our results clearly show that HDAd-Fsyn is capable of inducing serum neutralizing antibodies against RSV, indicating that HDAd-Fsyn has promise as a candidate RSV vaccine.

Long-lasting protective immunity is indispensable for a potential vaccine. McGinnes et al. explored the potential of virus-like particles (VLPs) as an RSV vaccine candidate and found that mice immunized with VLPs generated antibodies that had neutralizing activity up to 120 days[23]. We evaluated RSV-F-specific immunity and the duration of the immune response induced by a single dose of i.n. HDAd-Fsyn vaccine. We found that HDAd-Fsyn evoked RSV-F-specific serum neutralizing antibody responses lasting for at least 14 weeks. Two mechanisms may be responsible for the transgene-specific immunity: (i) HDAd vaccines can express the transgene durably *in vivo* and achieve a high level of transgene protein in infected dendritic cells (DCs) [13,24]; and (ii) HDAd vaccines can produce lower anti-Ad T-cell responses [12]. After i.n. RSV challenge, the lower virus titers in lung tissues from vaccinated mice demonstrated induction of efficient protective immunity compared with the control group.

## Conclusion

In summary, HDAd-Fsyn mucosal vaccine induced RSV-F-specific cellular and humoral immune responses and protected against subsequent i.n. RSV infection in a murine model. Our results suggest that HDAd has promise as a candidate i.n. RSV vaccine vector. Further development, such as co-expression of protective antigens or a combination with a DC-targeting strategy, might significantly enhance the efficacy of these vectors.

### Ethical approval

Animal experiments that is reported in the manuscript has been performed with the approval of the Committee on the Ethics of Animal Experiments of Beijing Jiaotong University (Permit number: 2010–0013). All surgeries were performed under sodium pentobarbital anesthesia, and all efforts were mase to minimize suffering.

## Competing interests

The authors declare that they have no competing interests.

## Authors’ contributions

JSH and FYH conceived the experiments. FYH, QW, JYY, HY, ZY and PXL performed the experiments. JSH and FYH analyzed the data. JSH, FYH and TH wrote the manuscript. All authors read and approved the final manuscript.
